# Liposuction Port-Site Protection: Necessity or Needless Expense?

**DOI:** 10.1093/asjof/ojae098

**Published:** 2024-11-26

**Authors:** Hunter R Moyer, Kayla M Sisson

## Abstract

**Background:**

Liposuction is the most common cosmetic plastic surgery procedure in the United States, and lipo-aspiration for fat grafting is gaining in popularity. The results are effective, but complications include seroma, contour irregularities, skin necrosis, and even death. Scarring, dehiscence, and infection at the port site, although minor, are a common and less-discussed problem. To date, no study has examined the local complication profile of patients treated with and without port-site protection.

**Objectives:**

To evaluate the efficacy of silicone port protectors to decrease local complications after liposuction.

**Methods:**

A retrospective review was performed on 60 consecutive patients treated for cosmetic liposuction or autologous fat transfer between August 2022 and March 2024. The first 30 patients underwent tumescent-based lipo-aspiration without port-site protectors and the following 30 with placement of a segment of suction tubing to protect the skin. Records were reviewed to determine patient demographics, amount of tumescent and aspirate, and complications at the port site.

**Results:**

Sixty patients completed the study. There were no significant differences in patient demographics and surgical data between groups. Ten patients in the unprotected group and 2 in the protected group experienced port-site complications (33.3% vs 6.7%, *P* = .0093). Significantly more patients in the unprotected group experienced wound dehiscence (*P* = .0095), and there was a trend toward more patients requiring steroid injections and excisions and reclosures (*P* = .088 and .167, respectively).

**Conclusions:**

In this cohort, patients treated with a port protection device suffered fewer local complications, requiring less wound management, steroid injections, and revisions.

**Level of Evidence: 3 (Therapeutic):**

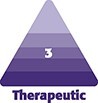

According to the American Society of Plastic Surgeons, liposuction held the top spot for the most common cosmetic surgical procedure in the United States in 2023.^1^ Autologous fat grafting has also grown exponentially with the potential to treat multiple cosmetic and reconstructive deformities.^2^ Both modalities require aspiration of fat through small skin incisions. As with any surgery, lipo-aspiration can result in complications, including seroma, contour irregularities, fat embolism, oil cysts, skin necrosis, and organ injury. Liposuction also has minor yet more common complications at the port site, including dehiscence, infection, hyperpigmentation, and hypertrophic scarring from the repetitive motion of the cannula against skin and subcutaneous tissues. These minor complications are not frequently quantified in the literature, although they directly affect patient satisfaction and can result in infection, multiple steroid injections, and scar revision procedures.

The use of skin protection strategies to reduce scarring is not novel and has previously been described in the literature. Friction and abrasive burns can be prevented through a variety of proposed means, such as making the port site larger, using lubricating gels, reducing the power of liposuction, as well as excising local tissue around the site prior to closure.^3^ Commercially available port-site protectors are available at a cost,^4^ and multiple patents have been issued.^5,6^ Alternatively, several authors have proposed the use of inexpensive substitutes, such as a truncated 1 mL plastic syringe,^7,8^ nasopharyngeal cannula,^9^ and modified nasogastric tubing.^10^

Although protecting the skin is intuitive, to date the literature has not proven a clinical benefit. The authors hypothesize that placing a silicone skin protector at the liposuction incision site will decrease local complications and lessen patient and surgeon burden.

## METHODS

This is a retrospective study of 60 consecutive patients who underwent cosmetic liposuction or lipo-aspiration for autologous fat grafting between August 2022 and March 2024 in Rapid City, SD. All patients were treated by a single surgeon at an outpatient surgery center through the tumescent liposuction technique without added energy modalities. No IRB was required at our facility, but patients consented for inclusion based on the National Research Act and Belmont Report. Patients were followed for a minimum of 3 months after surgery to assess both major complications and minor complications. A minor complication was defined as any instance of hypertrophic scarring, wound dehiscence, local infection, need for steroid injection, or revision at the port sites.

The first 30 patients underwent manual or suction-assisted lipectomy without placement of any skin-protecting device at the port site. Briefly, a #15 blade scalpel was used to make stab incisions at the desired entry point. In the nonprotection group, tumescent was infused to the desired volume, and liposuction was performed with a #4-26 Mercedes cannula or a #4-26 Basket cannula. Sterile, lubricating jelly was applied to the cannula repetitively. Closure was accomplished with 4-0 nylon interrupted sutures, and the site was dressed with mastisol, steri-strips, gauze, and occlusive tape.

**Figure 1.**
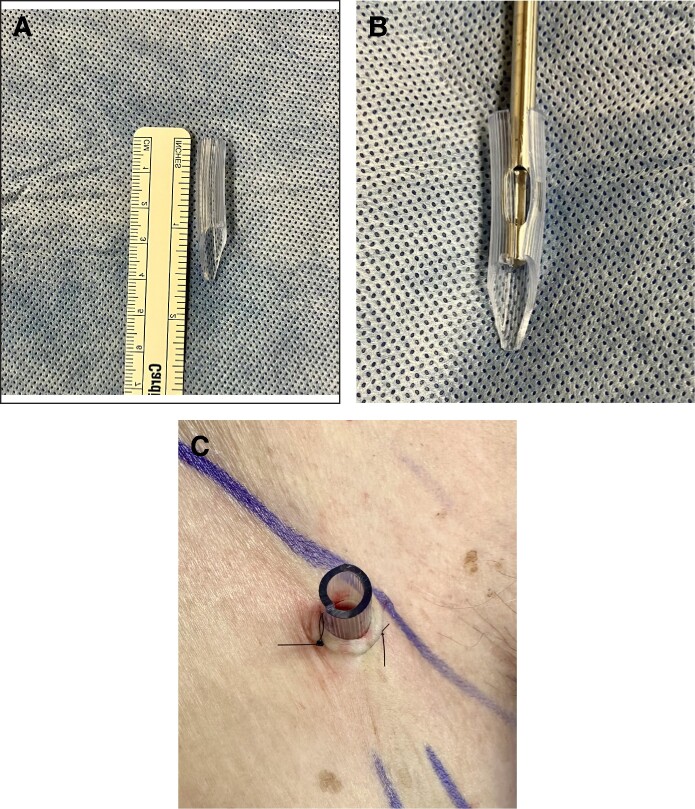
Port-site protector made from suction tubing with a slit along the axis to allow for larger cannulas (A and B) and secured to the skin with 4-0 nylon suture (C).

The second cohort of 30 patients underwent similar liposuction procedures but with the addition of placing a silicone protector at the port site. In this group, a section of sterile suction tubing was cut with Mayo scissors to 40 mm in length (Figure 1A). Each protector was cut with a slit along the axis to accommodate larger cannulas (Figure 1B) and tapered at 1 end for ease of entry into the skin and subcutaneous tissue. The incision site was bluntly dissected with tenotomy scissors, and the tubing was inserted and secured to the skin with two 4-0 Nylon interrupted sutures with ∼10 mm of tubing projecting from the skin (Figure 1C). Lubricating jelly was applied to the cannula at intervals. Once lipo-aspiration was complete, all stab incisions were closed with 4-0 Nylon interrupted sutures and dressed with mastisol, steri-strips, gauze, and occlusive tape.

Follow-up was performed by the operative surgeon, and patients were assessed for major complications as well as port-site complications. If required, steroid injections and scar revisions were performed by the operative surgeon in the clinic under local anesthesia. Information was pooled from clinic notes, operative notes, and follow-up encounters. The data were queried with Microsoft Excel (Microsoft Corporation, Redmond, WA) and statistics analyzed using StatPlus software (AnalystSoft, Alexandria, VA). A 2-tailed *t* test set for a Type I error of 5% (alpha = 0.05) was used to determine significance between data.

## RESULTS

All 60 patients completed the study with an average follow-up of 164 days (unprotected 173 days, protected 133 days, *P* = .061). The mean patient age was 49.4 years (range, 34-70 years) with no difference between groups (unprotected 50.3, protected 48.5, *P* = .50). The average BMI was 29.1 with similar numbers between cohorts (unprotected 28.9, protected 29.3, *P* = .75). Other comorbidities were matched between groups (Table 1).

**Table 1.** Patient Demographics

	Unprotected	Protected	*P*-value
Age (years)	50.3	48.5	.50
BMI	28.9	29.3	.75
Tobacco use	2	4	.39
Diabetes	2	4	.39

There were equal distributions of patients undergoing cosmetic liposuction vs fat grafting in both groups, and there was no difference in the overall tumescent infused (unprotected 1053.3 cc, protected 1148.3 cc, *P* = .50) nor fat aspirated between groups (unprotected 280.8 cc, protected 260.4 cc, *P* = .80). A majority of patients underwent liposuction to their abdomen (81.8% unprotected vs 70.5% protected, *P* = .32) with additional areas, including flanks, back, axillae, and knees. There were no major complications in either group (Table 2).

**Table 2.** Treatment Statistics

	Unprotected	Protected	*P*-value
Tumescent in (cc)	1053	1148	.50
Aspirate out (cc)	280.8	260.4	.80
Follow-up time (days)	173	133	.061

Ten of the 30 unprotected patients experienced a complication at the port site (33.3%). We noted 6 wounds that dehisced with prolonged drainage, 2 of which required excision and closure and 4 that healed by secondary intention. Five patients underwent serial steroid injections with a total of 9 treatments (average 1.8 injections per patient). Two of the steroid-injected patients went on to excision and closure for a total of 4 revisions (13.3%). Finally, there was 1 instance of superficial wound infection requiring oral antibiotics (Table 3).

**Table 3.** Complications

	Unprotected	Protected	*P*-value
Overall	10 (33.3%)	2 (6.67%)	.0093
Wound dehiscence	6 (20.0%)	0 (0%)	.0095
Steroid injections (pts)	5 (16.7%)	1 (3.33%)	.088
Steroid injections (#)	9	1	.0050
Wound infection	1 (3.33%)	0 (0%)	.32
Revision closure	4 (13.3%)	1 (3.33%)	.167

**Figure 2.**
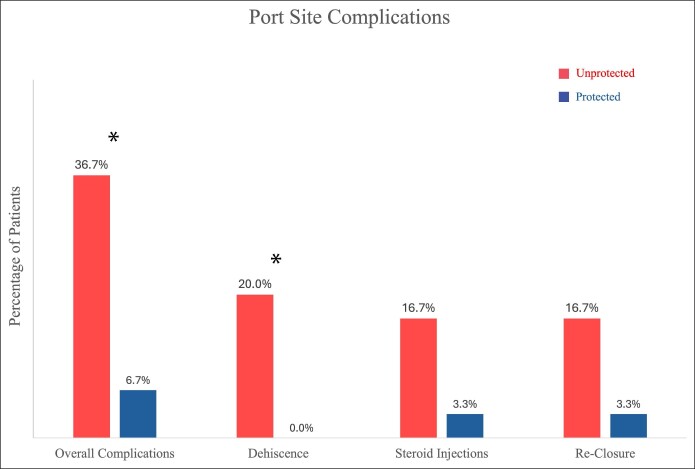
Complications. *p < 0.05.

Two of the 30 patients in the protected group experienced local complications (6.67%): 1 patient underwent a single steroid injection into the scar and deeper tissue, and another underwent excision and closure 98 days postoperatively. No patients developed a wound infection (Figure 2).

## DISCUSSION

**Figure 3.**
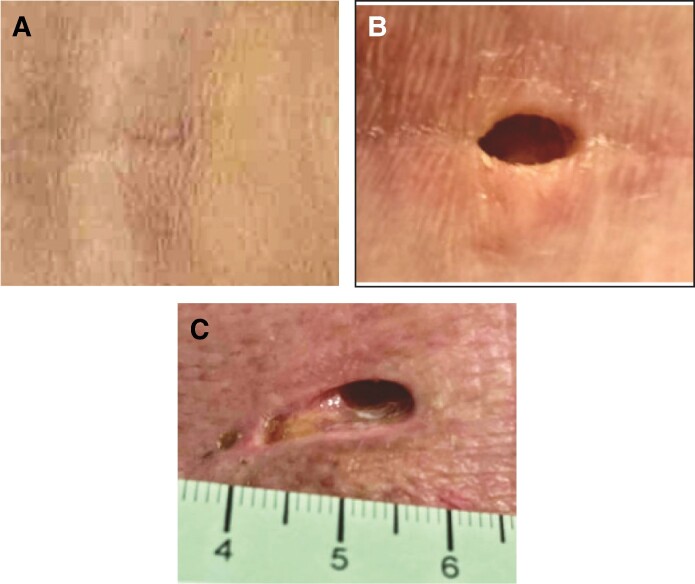
Postoperative images of port sites healed appropriately (A) and with dehiscence (B and C).

Multiple papers have shown patient satisfaction rates with aesthetic liposuction between 76% and 86%.^11-13^ Satisfaction is correlated with patient motivation, weight gain after surgery, postoperative discomfort, and complications. Complications are well documented, but problems with port-site healing and scarring, while understood, are less defined in the literature (Figure 3). Various techniques for closure of the stab incisions are described, including tissue glue, dissolvable sutures, and permanent sutures, and even excision of surrounding tissue and closure in a larger ellipse. No matter the closure technique, the skin and subcutaneous tissue are exposed to abrasive and thermal injury from the repetitive motion.^14^ The authors have used lubricating jelly to help reduce friction; however, the number of wound healing complications remained high.

The introduction of energy modalities to standard liposuction, such as ultrasound-, power-, and LASER-assisted devices, has increased the likelihood of skin injury.^15-17^ Thermal burns and scarring at port sites are not limited to liposuction procedures, because the authors of a recent study found a 54% thermal injury rate at port sites for robotic and laparoscopic transabdominal preperitoneal hernia repairs.^18^ The energy is primarily focused at the skin and subcutaneous site and is dispersed and dissipated in deeper tissue.

Standard suction tubing has an inner diameter of 4.8 mm and an outer diameter of 7.2 mm, making it ideal for most cannulas without enlarging the incision site. Although not measured precisely, we estimate that the addition of this port protector lengthened our incisions from 6 to 7.5 mm. The tubing is inexpensive, readily available, flexible, and easily cut to fit. We note the tubing is much more maneuverable than the harder plastic of a nasograstic tube or commercially available devices. For cosmetic liposuction, the tubing is added to the case cart at a cost of $1.87 at our surgery center, and for autologous fat grafting, there is no additional cost as we aspirated at the end of the case when suction tubing was no longer required and could be cut. Any number of protectors can be cut from 1 tubing, and although the cost of a 1 cc syringe^7,8^ is ∼$0.78, one will be needed for each site. Nasogastric tubing is quoted at a cost of $8 per site by Hui and Chui^10^ and the devices by Black and Black Surgical (Atlanta, GA) and Exo-Port run $58 and $40 per port, respectively. The protectors should be measured to a length of 40 mm to allow for 30 mm of depth to protect the skin and subcutaneous tissue, because we have experienced both skin and deeper subcutaneous scar formation in unprotected sites.

There were no major complications in this cohort, and there was a significant decrease in all local complications and wound dehiscence. We noted a trend toward fewer patients requiring scar revision and steroid injections for hypertrophic scars, but a significant decrease in the overall number of steroid injections performed (9 vs 1, *P* = .005). The overall cost savings are difficult to determine, but the burden on the clinic, surgeon, and patient is clearly reduced with less complications.

The current study is limited by the relatively small patient population, the use of a single modality of aspiration (no added energy), the potential surgeon bias in evaluating results, and incongruent follow-up time between groups. As most wound healing complications occur in the first 3 months after surgery, we do not feel we have missed additional scarring or infections in the protected group despite a trend toward a significantly shorter follow-up time. Additionally, energy-assisted techniques have a higher complication profile, and one could actually expect a more significant difference should they be included. To limit surgeon bias, we chose endpoints of wound dehiscence, need for steroid injections, and reclosure as objective measures instead of subjective scar evaluations. Finally, although the numbers are small, we found a significant improvement in port-protected patients.

## CONCLUSIONS

This is the first study to quantify the difference in complications between protected and unprotected port sites during liposuction procedures. In this cohort, patients treated with a port protection device suffered significantly fewer local complications, requiring less wound management, steroid injections, and scar revisions.
